# Impact of adult attachment styles on health behaviors among youngsters

**DOI:** 10.1192/j.eurpsy.2021.1239

**Published:** 2021-08-13

**Authors:** S. Masood, D.S. Ali

**Affiliations:** Department Of Psychology, University of Karachi, Karachi, Pakistan

**Keywords:** Adult Attachment Style, Health Behavior, Attachment Style

## Abstract

**Introduction:**

Attachment styles play important role in managing health behavior. It has been observed in researches that attachment style have relationship with health behavior (Schlack, 2003). If attachment styles are left the way they form habitually it can be harmful, in a study with diabetic patients, people with avoidant attachment style were expected to die within 5 years of disease diagnosis (Ciechanowski et al., 2010).

**Objectives:**

To assess effect of adult attachment styles on health behavior?

**Methods:**

Sample comprised of 300 university students from different private and government universities of Karachi with age range 18 – 35 years. Assessment tools used are relationship questionnaire and wellness behavior inventory scale. Relationship questionnaire is used to identify the dimension of attachment style (Bartholomew & Horowitz, 1991). Wellness behavior inventory was used to identify consistency of healthy behavior activities done on regularly basis (Sirois, 2001). Statistical tests used for descriptive analysis were frequency and percentage and for inferential statistics regression analysis test was used.

**Results:**

According to the attachment styles A, B, C and D most of the study participants fell in healthy weight range, a few were in obese range which is considered unhealthy. Result of regression analysis estimated there is no effect of attachment style on health behavior as p-value was greater than 0.05.

**Conclusions:**

Attachment style is not a good predictor of health behavior solely. As per a few researches in order to study impact of attachment styles other mediating variables that can have effect on health behavior should also be observed such as self-esteem.

